# Acute Dysphasia and Reversible Cognitive Decline in a Patient with Probable Cerebral Amyloid Angiopathy-Related Inflammation

**DOI:** 10.1155/2015/189581

**Published:** 2015-03-02

**Authors:** Louise Rigney, Dale Sebire, Dennis Cordato

**Affiliations:** Neurophysiology Department, Liverpool Hospital and South Western Clinical School, University of New South Wales, Elizabeth Street, Liverpool, NSW 1871, Australia

## Abstract

Cerebral amyloid angiopathy related inflammation (CAAri) is becoming increasingly recognised as a subset of cerebral amyloid angiopathy (CAA). CAAri generally presents with subacute cognitive decline, headaches, seizures, behavioral changes, and focal neurological deficits. We describe a patient who developed acute dysphasia and reversible cognitive decline due to probable CAAri. CT brain showed bilateral vasogenic edema in the cerebral hemispheres, predominantly involving the parietal and temporal lobes, left greater than right without enhancement. Magnetic resonance brain imaging showed extensive multifocal areas of subcortical white matter T2 hyperintensity in the frontal and temporal regions with associated mass effect, negligible enhancement, and multiple foci of microhemorrhage on susceptibility weighted imaging sequences consistent with a diagnosis of probable CAAri. She responded dramatically to a course of intravenous methylprednisolone followed by further immunosuppression with pulse intravenous cyclophosphamide. Her dysphasia resolved within 5 days of intravenous methylprednisolone therapy. Her MMSE improved from 11/30 at day 5 of admission to 28/30 at 6-month follow-up. The notable features of our case were the unusual CT findings, which were inconsistent with stroke and diagnostic utility of susceptibility-weighted magnetic resonance imaging in confirming the diagnosis which allowed for prompt institution of immunosuppression.

## 1. Introduction

Cerebral amyloid angiopathy (CAA) is defined by the deposition of amyloid proteins within leptomeningeal and cortical arteries, arterioles, capillaries, and, rarely, veins [1]. Sporadic CAA increases with age, rarely being present before the age of 50 and increasing in prevalence to more than 50% in nonagenarians [1]. The clinical spectrum includes lobar intracranial hemorrhage, subarachnoid hemorrhage, ischemic infarction, focal seizures, transient ischemic attacks, positive visual phenomena similar to migraine, cognitive impairment, and dementia [1]. CAA-related inflammation (CAAri) has been recently recognised as a rare mechanism by which CAA causes dementia [1–5]. The primary manifestation of noninflammatory CAA is hemorrhagic stroke [3]. In contrast, CAAri most often manifests as subacute cognitive decline with or without acute confusion and seizures [1–7]. Aphasia without hemorrhage is also described mostly as an associated symptom and less commonly as a predominant feature [2, 3].

We report a 65-year-old female who presented with acute aphasia and reversible cognitive decline due to probable CAAri.

## 2. Case Report

A 65-year-old previously independent right-handed female presented with a six-day history of altered mental status. Her past medical history included hypertension, type 2 diabetes mellitus, and hyperlipidaemia. Her medications consisted of Atorvastatin 20 mg daily, Fosinopril 20 mg daily, Sitagliptin 50 mg twice daily, and Metformin 500 mg twice daily.

Examination revealed severe expressive and receptive aphasia and mild pyramidal weakness (Medical Research Council grade 4/5) of the right upper and lower limbs. The rest of the neurological examination was normal.

**Figure 1:**
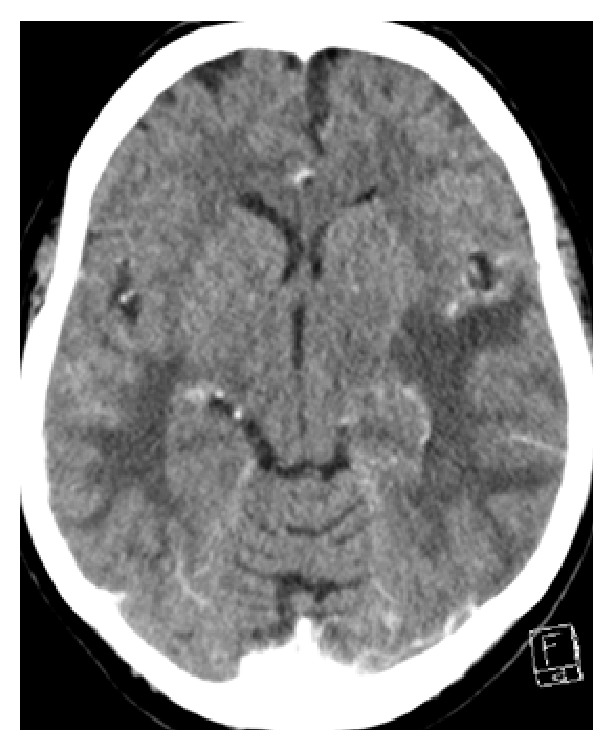
Contrast enhanced CT brain showing bilateral vasogenic edema of the parietal and temporal lobes.

Routine blood tests and autoimmune, thrombophilic, and vasculitic screens were normal. Contrast-enhanced CT brain showed bilateral vasogenic edema in the cerebral hemispheres, predominantly involving the parietal and temporal lobes, left greater than right. There was no enhancement or vascular abnormalities (Figure 1).

CSF showed a lymphocytic pleocytosis [WCC 19 × 10^6^/L (70% mononuclear), RCC 4110 × 10^6^/L], elevated protein (1.94 g/L), and elevated glucose (5.5 mmol/L) (serum glucose 8.9). Testing for bacterial, viral, fungal, and cryptococcal pathogens was negative. She was homozygous for the Apolipoprotein E (APOE) e4 genotype.

EEG showed generalised slow wave activity with superimposed focal left hemisphere slowing.

**Figure 2:**
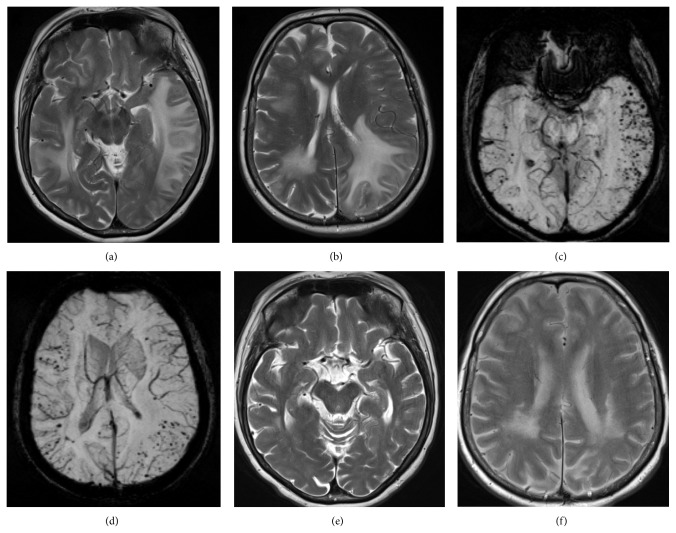
(a and b) T2 TSE images showing multifocal areas of white matter T2 hyperintensity. (c and d) Susceptibility weighted images showing multiple foci of microhemorrhage. (e and f) Follow-up T2 TSE images at 6 months showing significant interval improvement of white matter hyperintensity.

Magnetic resonance imaging of the brain (MRI) showed extensive multifocal areas of subcortical white matter T2 hyperintensity in the frontal and temporal regions with associated mass effect, negligible enhancement, and multiple foci of microhemorrhage on susceptibility weighted imaging sequences consistent with CAAri (Figures 2(a)–2(d)).

She was treated with IV methylprednisolone 500 mg/day for 5 days followed by oral prednisolone 50 mg daily. Her dysphasia significantly improved during IV methylprednisolone therapy. At day 5, her dysphasia had resolved. Her mini-mental state examination score at that time was 11/30. IV cyclophosphamide was initiated on day 21 after admission (6 × fortnightly 500 mg doses).

Repeat MRI at day 12 showed mild resolution of white matter T2 hyperintensities within the left basal ganglia.

Six weeks after initial presentation, she was at home, independent in all activities of daily living with a repeat MMSE score of 23/30. The oral prednisolone was gradually tapered over 3 months to a maintenance dose of 10 mg daily. At last follow-up, 6-months after discharge, her MMSE score had further improved to 28/30 and MRI, at this time demonstrating significant reduction in white matter T2 hyperintensities (Figures 2(e)-2(f)).

## 3. Discussion

We report a rare case of acute onset aphasia and reversible cognitive decline due to CAAri. CAAri is a rare subset of CAA. Whereas hemorrhagic stroke is a common manifestation of CAA, CAAri generally presents with headaches, seizures, subacute cognitive decline, behavioral changes, and focal neurological deficits [1–3]. Aphasia is a less common presentation of CAAri [1–3]. Sakaguchi et al. reported that higher brain dysfunction without encephalopathy or dementia was seen in 10 of 64 (15%) published cases, with aphasia as the most frequent symptom (9%) [7]. In another review of 69 cases of pathologically confirmed CAAri, aphasia was as an associated feature in 26% [3].

Characteristic MRI findings in CAAri include large confluent or patchy areas of asymmetric T2 weighted or FLAIR white matter hyperintensities, as seen in our patient (Figures 2(a)–2(d)), with or without patchy contrast enhancement of leptomeninges or parenchymal lesions. Cortical-subcortical microhemorrhaging found on T2^*^gradient echo or susceptibility-weighted imaging confirmed the clinical diagnosis in our case without need for biopsy confirmation [3]. Microhemorrhaging on T2-weighted gradient-echo sequence images was absent in 2 of 13 (13%) previously reported CAAri cases [7]. A biopsy should be strongly considered in such patients [7].

It has been postulated that CAAri is mediated by autoantibodies to vascular Amyloid *β* (A*β*)—the same 1–42 amino acid A*β* peptide seen in Alzheimer's disease (AD) plaques. This is supported by studies that show CSF A*β* antibodies are elevated in acute CAAri and reduce corresponding with clinical and radiological improvements [4, 6, 8]. CSF analysis in our patient showed pleocytosis with raised protein. We were unable to test for CSF A*β* antibodies at our institution.

Recent immunotherapeutic approaches directed at reducing cerebral A*β* burden in AD have been found to cause vasogenic edema which is clinically, radiographically and pathologically similar to CAAri. Immunotherapy-related vasogenic edema may represent a treatment-induced equivalent to CAAri, directed against amyloid that has moved from brain parenchyma into and out of the cerebral vasculature [5].

Our patient was homozygous for the APOE e4 allele. Both CAAri and immunotherapy-related inflammation are strongly associated with APOE e4 allele dose, suggesting that the e4 isoform may play a specific role in promoting an inflammatory response to CAA [2, 5, 6].

Recognition and diagnosis of CAAri are important because it is potentially amenable to treatment with immunosuppression [2, 3, 9]. Our patient rapidly improved after initial treatment with high dose corticosteroids. Pulse intravenous cyclophosphamide was introduced as adjunct therapy at 3 weeks. Other adjunct immunosuppressants that have been used in previously published case series include methotrexate and mycophenolate mofetil [3]. The optimum duration of treatment is unknown [3]. The treatment duration in our patient will be based on clinical and radiological response [3] but immunosuppressant therapy is likely to be gradually withdrawn within 12 months. In previously published series, patients who have relapsed or experienced recurrence of symptoms with reduction or cessation of therapy have generally responded to reinstatement of therapy [3].

In conclusion, we describe a patient with acute aphasia and reversible cognitive decline due to CAAri. The notable features of our case were the unusual CT findings, which were inconsistent with stroke, and diagnostic utility of susceptibility-weighted imaging in confirming the diagnosis which allowed for prompt institution of immunosuppression.
